# Multidrug Resistance of *Escherichia coli* Strains Isolated From Bovine Feces and Carcasses in Northeast Mexico

**DOI:** 10.3389/fvets.2021.643802

**Published:** 2021-04-23

**Authors:** Ana V. Martínez-Vázquez, Jose Vázquez-Villanueva, Luis M. Leyva-Zapata, Hugo Barrios-García, Gildardo Rivera, Virgilio Bocanegra-García

**Affiliations:** ^1^Centro de Biotecnología Genómica, Instituto Politécnico Nacional, Reynosa, Mexico; ^2^Facultad de Medicina Veterinaria y Zootecnia, Universidad Autónoma de Tamaulipas, Ciudad Victoria, Mexico

**Keywords:** bovine, antimicrobial resistance, MDR, *Escherichia coli*, ESBL

## Abstract

In this work, the antimicrobial resistance profile of *Escherichia coli* strains (*n* = 248) isolated from bovine feces and carcass samples from Tamaulipas, Mexico, was evaluated. Susceptibility to 12 antibiotics conventionally used in human and veterinary treatments was determined according to Clinical and Laboratory Standards Institute guidelines. Genes encoding resistance to tetracycline (*tetA* and *tetB*), streptomycin (*str*A), aminoglycoside (*aad*A), and β-lactamase (*bla*_TEM_ and *bla*_SHV_) were investigated by PCR. Also, *stx*1, *stx*2, *eae, bfp*, and *hly*A encoding virulence factors were determined. Of the isolates, 85.9% were confirmed as *E. coli* strains. Among the 213 *E. coli* isolates tested, 94.8% (202/213) showed resistance for at least one antimicrobial, mainly ampicillin (83.0%; 177/213), cephalothin (76.0%; 162/213), and tetracyclines (69.0%; 147/213). In all the other antibiotics tested, the resistance percentage was below 36%. A multidrug-resistant phenotype was found in 72.7% of the tested strains. The presence of the *tet* gene (*tet*A or *tet*B) was detected in 43.1% of the isolates, the *str*A gene in 17.3%, and *aad*A1 in 51.6%. The *bla*_TEM_ and *bla*_SHV_ genes were found in 10.3 and 0.4% of the isolates, respectively. *stx*1 was detected in 4.2% of isolates, *stx*2 in 7.0, and *hly*A in 2.8%. The virulence genes, *eae* and *bfp*, were not detected in any strain. These results indicate that Tamaulipas food products of bovine origin can be a source of multiresistant *E. coli* strains for the environment and exposure for consumers.

## Introduction

Antimicrobial resistance (AMR) is a complex multifactorial process driven by numerous and varied factors (1). A causal relationship has been demonstrated with the increased use of antimicrobials in veterinary medicine for treating and preventing disease and for growth promotion (2). Antibiotic-resistant bacteria can be transferred directly from animals to humans and can spread to the soil, food, and groundwater by the application of manure to agricultural fields (3–5). The emergence of AMR in food-producing animals is of concern since these zoonotic bacteria pose a risk to human health *via* the food chain (6, 7). Resistant proportions in the sentinel organism *Escherichia coli* are often referred to as “prevalence” of resistance (8). *E. coli* is known as a dangerous pathogen to human health. It contributes to the dissemination of AMR, and it is a good indicator of transmission pathways because it is ubiquitous, shares the same niche as other enteric pathogens, and can be transferred by the same route (7, 9, 10). *E. coli* strains can colonize cattle at any age (11), and bovine carriers are often asymptomatic. Consequently, these enteric pathogens, when shed in the feces, are a source of contamination of water and food products (12). Analysis of antibiotic resistance in microbial isolates from agricultural and other environments could help us recognize resistance gene reservoirs that may be present in bacterial populations associated with food production animals (13). Monitoring of AMR in *E. coli* may provide information on antimicrobial abuse and the dynamics of transmission and development of AMR pathogens. Only a few reports from México about the AMR profiles of *E. coli* isolates in food-producing animals are available. Amezquita et al. (14), in northwestern Mexico, examined the AMR profiles of Shiga toxin-producing *E. coli* (STEC) O157 and non-O157 from cattle, sheep, and poultry. Multidrug resistance profiles were identified in 42% of the non-susceptible STEC strains. However, in northeast Mexico, no information has been generated regarding the possible multidrug resistance in cattle (feces or carcasses) and its potential risk for human health. The objective of this work was to investigate the AMR profile in *E. coli* isolated from cattle in northeast Mexico.

## Materials and Methods

### Sample Collection

Samples were collected from 124 cattle from a bovine slaughterhouse located in the center of the state of Tamaulipas, Mexico. This slaughterhouse receives cattle from nearby municipalities. One specimen was randomly selected per farm for the fecal sample and the carcass sample later to be taken.

### Fecal Samples

A total of 124 bovine fecal samples were collected by direct rectal retrieval using disposable gloves. Each glove was inverted and sealed immediately after collection.

### Carcass Samples

A total of 124 carcass samples were collected by sterile sponge from each carcass 12 h after evisceration: a minimum of 100 cm^2^ from inside the round area from the navel–plate–brisket–foreshank areas. All carcass samples were collected according to the national regulations described in NOM-SSA1-1994 (15). All samples were placed in separate sterile plastic bags to prevent cross-contamination, labeled, and transported in ice to the laboratory.

### Isolation and Identification of *E. coli*

Twenty-five grams of each sample was enriched by inoculation in 225 ml of peptone water. They were then homogenized and incubated at 37°C. After 24 h, the samples were plated onto eosin methylene blue and were incubated at 37°C for 18–24 h. Six colonies of presumptive *E. coli* strains were randomly picked from each sample, and each was subsequently transferred separately to tryptic soy agar and incubated for 24 h at 37°C to obtain a pure culture. All suspect colonies were confirmed by biochemical identification, including the sugar fermentation test, the indole production test, and the citrate test (16).

### Antimicrobial Susceptibility Testing

Antimicrobial susceptibility was tested by the agar disc diffusion method, following the Clinical and Laboratory Standards Institute guidelines (17). The antimicrobials used were tetracycline (TE; 30 μg), ampicillin (AM; 10 μg), levofloxacin (LEV; 5 μg), cephalothin (CF; 30 μg), cefotaxime (30 μg), gentamicin (GE; 10 μg), cefepime (30 μg), trimethoprim/sulfamethoxazole (25 μg), netilmicin (NET, 30 μg), amikacin (30 μg), ceftriaxone (30 μg), and streptomycin (STR, 30 μg) (Sensi-disc BBL). These antimicrobials represent the major classes of antimicrobial drugs relevant in both veterinary and human medicine. Each *E. coli* strain colony was evaluated based on the diameter of the clear inhibition zone, and the results were interpreted following the criteria provided by the CLSI. *E. coli* ATCC 25922 was used as a control.

### Antibiotic-Resistant Gene Detection

Bacterial DNA was obtained from a pure culture on tryptic soy agar (BD Becton Dickinson & Co., Cuautitlán Izcalli, Mexico) by lysis of a bacterial cell suspension at 95°C for 15 min, followed by centrifugation at 13,000 × g for 3 min. Six antibiotic resistance genes were screened by PCR: genes encoding tetracycline efflux pump (*tet*A and *tet*B), streptomycin phosphotransferases (*str*A), aminoglycoside adenyltransferase (*aad*A), and beta-lactamase resistance (*bla*_TEM_ and *bla*_SHV_) (18–20). PCR conditions were performed in a total volume of 25 μl containing 1× buffer, 25 mM MgCl_2_, 10 mM dNTPs (Bioline), 10 mM of each primer, 5 U of Taq polymerase (Uniparts), and sterile water. PCR amplification was conducted following these conditions: initial denaturation at 95°C for 7 min, followed by 30 cycles of denaturation at 95°C for 45 s, annealing at 42 at 59°C for 45 s, and extension at 72°C for 45 s and a final cycle of amplification at 72°C for 7 min. The PCR products were analyzed by gel electrophoresis in 2.5% agarose (Sigma Aldrich, Germany), followed by visualization on a transilluminator.

### Detection of Virulence Factors

The detection of virulence factor genes was performed according to the method described by Canizalez et al. (21). PCR was carried out using target gene-specific primers, including shiga toxin (*stx*1 and *stx*2), intimin (*eae*A), bundle-forming pilus (*bfp*), and enterohemolysin A (*hly*A). The PCR reaction mixture contained buffer 1X, MgCl_2_ 25 mM, dNTPs 10 mM, primer 10 mM, and Taq DNA polymerase 5 U. The volume of this mix was adjusted to 25 μl with sterile water. The PCR amplification conditions were as follows: initial denaturation at 95°C for 1 min, followed by 30 cycles of denaturation at 95°C for 45 s, annealing at 54–60°C for 45 s, and extension at 72°C for 45 s and a final cycle of amplification at 72°C for 7 min. Verification of PCR products was performed in horizontal electrophoresis using 2.5% agarose gel with 0.5× TBE and Sybr gold at 100 V for 45 min. A molecular marker was run concurrently (100 pb Promega). The negative control consisted of all contents of the reaction mixture excluding template DNA, which was substituted with 1 μl sterile water. The DNA bands were visualized and photographed under UV light.

## Results

### Isolation and Identification of *E. coli*

Out of 248 samples tested, *E. coli* strains were detected in 85.9% (213/248); 87.9% (109/124) were confirmed as *E. coli* strains in fecal samples, and 83.8% (104/124) were from carcass samples (Table 1).

**Table 1:** Prevalence of phenotypical antimicrobial resistance in *E. coli* recovered from bovine feces and carcasses.

**Antibiotic**	**Bovine feces, *n* = 109 (%)**	**Bovine carcasses, *n* = 104 (%)**	**Total *n* = 213 (%)**
Streptomycin	44 (40.3)	20 (19.2)	64 (30.0)
Cefepime	66 (60.5)	0	66 (30.9)
Gentamicin	17 (15.5)	1 (0.9)	18 (8.4)
Cefotaxime	48 (44.0)	1 (0.9)	49 (23.0)
Trimethoprim/ sulfamethoxazole	41 (37.6)	11 (10.5)	52 (24.4)
Tetracycline	78 (71.5)	69 (66.3)	147 (69.0)
Ampicillin	75 (68.8)	102 (98.0)	177 (83.0)
Levofloxacin	11 (10.0)	2 (1.9)	13 (6.1)
Cephalothin	81 (74.3)	81 (77.8)	162 (76.0)
Netilmicin	8 (7.3)	8 (7.6)	16 (7.5)
Amikacin	13 (11.9)	11 (10.5)	24 (11.2)
Ceftriaxone	33 (30.2)	25 (24.0)	58 (27.2)
Antimicrobial resistance to at least one antimicrobial	99 (90.8)	103 (99.0)	202 (94.8)
Multidrug-resistant	73 (66.9)	82 (78.8)	155 (72.7)

### Antimicrobial Susceptibility Testing

Among the 213 *E. coli* isolates tested, 94.8% (202/213) showed resistance for at least one antimicrobial, mainly AM (83.0%; 177/213), CF (76.0%; 162/213), and TE (69.0%; 147/213) (Table 1). The percentage of resistance to the other antimicrobial agents was in all cases below 36%. The results showed resistance to AM in 68.8% of fecal strains (75/109) and 98.0% of carcass strains (102/104). Phenotypic resistance to CF *E. coli* was confirmed in 74.3% of fecal isolates (81/109) and 77.8% of carcass isolates (81/104). The isolates that showed resistance to TE were 71.5% of fecal strains (78/109) and 66.3% of carcass strains (69/104). The percentage of sensitivity to LEV (90.6%; 193/213), GE (85.9%; 183/213), and NET (80.7%; 172/213) shown in this work was higher than 80%. Fifty-seven antibiogram resistance patterns were detected in this study (46 fecal and 11 carcasses; Table 2). The detection of 72.7% (155/213) strains which showed a MDR phenotype (104 fecal and 82 carcasses) is remarkable.

**Table 2:** Some resistance profiles of bovine feces and carcasses.

**Sample**	**Number of antibiotics**	**Patterns of resistance phenotypes**												**Isolates**
		**AM**	**CF**	**TE**	**STR**	**CRO**	**STX**	**CTX**	**FEP**	**AK**	**NET**	**GE**	**LEV**	
Bovine feces	3	AM	CF		STR									2
	3	AM	CF					CTX						2
	3		CF	TE	STR									3
	3		CF	TE					FEP					2
	4	AM	CF	TE	STR									3
	4	AM		TE	STR	CRO								1
	5	AM	CF	TE		CRO		CTX						2
	5	AM	CF			CRO		CTX	FEP					2
	6	AM	CF	TE	STR			CTX	FEP					2
	6	AM	CF	TE	STR		STX	CTX						2
	6	AM	CF			CRO	STX	CTX	FEP					4
	7	AM	CF	TE		CRO	STX	CTX	FEP					3
	7		CF	TE	STR	CRO	STX	CTX	FEP		NET			2
	8	AM	CF	TE	STR	CRO	STX	CTX	FEP					3
Bovine carcasses	3		CF	TE		CRO								9
	4			TE		CRO								2
	5	AM	CF	TE		CRO	SXT							24
	6	AM		TE										2
	6	AM	CF	TE	STR	CRO	SXT			AK		GE		6
	6	AM		TE									LEV	1
	6	AM	CF	TE	STR	CRO	SXT				NET			8
	6	AM	CF	TE		CRO				AK				2
	7	AM	CF	TE	STR	CRO	SXT		FEP	AK				2
	7	AM		TE	STR	CRO	SXT							3
	7	AM	CF	TE		CRO		CTX		AK			LEV	1

### Antibiotic-Resistant Gene Detection

Of the isolates, 19.2% (41/213) were negative for all the resistant genes tested. The *tet*(A) and *tet*(B) genes were detected in 43.1% of isolates (92/213; 35 fecal and 57 carcasses). Both *tet* genes were detected in 12.6% (27/213) of isolates (17 fecal and 10 carcasses). The *str*A and *aad*A1 genes were detected in 17.3% (37/213; eight fecal and 29 carcasses) and 51.6% (110/213; six fecal and 104 carcasses) of isolates, respectively. β-Lactamic genes were detected in 10.3% (22/213; 18 fecal and four carcasses) from *bla*_TEM_ and in 0.4% (1/213; one fecal and zero carcasses) from *bla*_SHV_ (Table 3).

**Table 3:** Distribution of resistance genes in *Escherichia coli* isolates from cattle.

**Sample types**	**Prevalence of *E. coli***	**β-Lactamic resistance**				**Tetracycline resistance**			**Streptomycin resistance**		
		**Phenotypic**		**Genotypic**		**Phenotypic**	**Genotypic**		**Phenotypic**	**Genotypic**	
		**AM**	**CF**	***bla***_**TEM**_	***bla***_**SHV**_	**TE**	***tet*** **(A)**	***tet*** **(B)**	**STR**	***str*****A**	***aad*****A-IV**
Fecal	109	75/109 (68.8%)	81/109 (74.3%)	18/109 (16.5%)	1/109 (0.9%)	78/109 (71.5%)	27/109 (24.7%)	8/109 (7.3%)	44/109 (40.3%)	8/109 (7.3%)	6/109 (5.5%)
Carcasses	104	102/104 (98.0%)	81/104 (77.8%)	4/104 (3.8%)	0/104 (0%)	69/104 (66.3%)	29/104 (27.8%)	28/104 (26.9%)	20/104 (19.2%)	29/104 (27.8%)	104/104 (100%)
Total	213	177/213 (83.0%)	162/213 (76.0%)	22/213 (10.3%)	1/213 (0.4%)	147/213 (69.0%)	56/213 (26.2%)	36/213 (16.9%)	64/213 (30.0%)	37/213 (17.3%)	110/213 (51.6%)

### Detection of Virulence Factors

Of the isolates, 14.0% (30/213) showed one or two genes encoding a virulence factor (*stx*1, *stx*2, or *hly*A). The *stx* genes were carried by 11.2% (24/213; 12 fecal and 12 carcasses) of the studied isolates. From these *stx* positives, 4.2% (9/213; three fecal and six carcasses) were *stx*1, and 7.0% (15/213; nine fecal and six carcasses) were *stx*2. Only three strains showed both genes *stx*1 and *stx*2 (1.4%; 3/213). However, *hly*A was detected in only 2.8% (6/213; four fecal and two carcasses). None of the isolates contained the virulence genes *eae*A and *bfp*.

## Discussion

Previous studies show that antimicrobial resistance varies widely depending on the countries or animal from which the microorganisms have been isolated. In this study, *E. coli* strains with resistance to AM (83.0%), CF (76.0%), and TE (69.0%) were the most prevalent. Overall, other studies with *E. coli* strains from bovine samples showed a range of antimicrobial resistance to AM between 6.0 and 95.7% and to TE between 10.6 and 95% (8, 22–26). Globally, TE and AM are widely used in animal production, disease prevention, and growth promotion. Their combined resistance is often due to the colocation of different determinants in the same mobile genetic elements (plasmids, transposons, and/or integrons), and this has contributed to the selection of multidrug-resistant isolates worldwide (23). Fifty-seven antibiogram patterns of resistance were detected in this study (46 fecal and 11 carcasses) (Table 2). In the current study, an overall prevalence of MDR among the isolates (72.7%; Table 1) was high compared to previous studies worldwide, for example, an MDR of 44.4% in Egypt (27), 69.0% in Portugal (28), 56.0% in France (29), 13.7% in South Africa (30), and 37.1% in Jordan (24). In Mexico, an AMR study in the northwest reported an MDR of 42% (14). This might be due to inappropriate or excessive use of antibiotics for therapeutic and prophylactic treatment (31, 32). These results suggest that bovines may represent an important reservoir of antibiotic-resistant bacteria, be a route of spread, and represent a health risk. Of the 147 *E. coli* strains with phenotypic resistance to TE, 38.0% (56/147) showed the gene *tet*A and 24.4% (36/147) the gene *tet*B. While the *tet*A gene had a similar prevalence in both types of samples (fecal, 27/109 and carcasses, 29/104) for the *tet*B gene, the prevalence was markedly higher in carcass samples compared to that in fecal samples (fecal, 8/109 and carcasses, 28/104). Of the 64 strains with phenotypic resistance to STR, 57.8% (37/64) have the resistance gene *str*A and 100% (64/64) the resistance gene *aad*A-IV. The 46 strains identified with the *aad*A-IV gene did not show phenotypic resistance to STR (Table 3). These resistance genes may be transferable from *E. coli* to other bacteria in the gut and subsequently disseminated into the environment. The virulence factors of *E. coli* strains were frequently associated with MDR. In the association between antimicrobial resistance and virulence factors, the most remarkable was resistance to AM, CF, TE, and STR in all strains with *stx*1 virulence factors (9/9); 100% (15/15) of strains *stx*2 showed resistance to at least one antimicrobial. Of these, 40% (6/15) showed resistance to four. One strain harboring *hly*A was not resistant to any antibiotic tested (6.6%; 1/15), and the rest of the *hly*A-harboring strains had antimicrobial resistance to two or more antibiotics (93.3%; 14/15). *E. coli* harboring *stx* and *eae* genes might represent a high zoonotic risk, as these genes are often found in human pathogenic enterohemorrhagic *E. coli* strains (26, 33, 34). In this study, the *stx* genes and the *hlyA* gene were present in our isolates, whereas *aea* and *bfp* were not detected. The gene *stx*2 codes for a potent toxin, often associated with the development of hemorrhagic colitis and hemolytic uremic syndrome in humans and was frequently associated with more severe human diseases than *stx1* (26, 35).

## Conclusions

In this study, we detected a high prevalence of *E. coli* strains with resistance (82.0%) and MDR (72.7%) with AM, CF, and TE being the least effective antibiotics. Although the prevalence of virulence factors was low, the strains harboring the virulence factors were frequently associated with MDR. These results suggest that bovines may represent an important reservoir of antibiotic-resistant bacteria and show the need to enforce the regulations in the use of antimicrobials in food-producing animals.

## Data Availability Statement

The raw data supporting the conclusions of this article will be made available by the authors, without undue reservation.

## Author Contributions

AM-V and JV-V participated in samples processing and laboratory analysis. LL-Z participated in samples acquisition and processing. HB-G and GR participated in data analysis and first draft. VB-G participated in study design and revised and approved the final manuscript. All authors contributed to the article and approved the submitted version.

## Conflict of Interest

The authors declare that the research was conducted in the absence of any commercial or financial relationships that could be construed as a potential conflict of interest.
